# The Role of Exogenous microRNAs on Human Health: The Plant–Human Trans-Kingdom Hypothesis

**DOI:** 10.3390/nu16213658

**Published:** 2024-10-28

**Authors:** Emanuela Pasculli, Raffaella Maria Gadaleta, Maria Arconzo, Marica Cariello, Antonio Moschetta

**Affiliations:** 1 Department of Interdisciplinary Medicine, University of Bari “Aldo Moro”, 70124 Bari, Italy; emanuela.pasculli@uniba.it (E.P.); raffaella.gadaleta@uniba.it (R.M.G.); maria.arconzo@uniba.it (M.A.); 2 INBB National Institute for Biostructure and Biosystems, Viale delle Medaglie d’Oro 305, 00136 Rome, Italy

**Keywords:** microRNA, trans-kingdom, plant miRNA, human miRNA, nutrition, human diseases

## Abstract

MicroRNAs (miRNAs) are small, endogenous, single-stranded RNAs that act on gene silencing at the post-transcriptional level by binding to a target messenger RNA (mRNA), leading to its degradation or inhibiting translation into functional proteins. The key role of miRNAs in development, proliferation, differentiation andapoptosis has been deeply investigated, revealing that deregulation in their expression is critical in various diseases, such as metabolic disorders and cancer. Since these small molecules initially evolved as a mechanism of protection against viruses and transposable elements, the fascinating hypothesis that they can move between organisms both of the same or different species has been postulated. Trans-kingdom is the term used to define the migration that occurs between species. This mechanism has been well analyzed between plants and their pests, in order to boost defense and increase pathogenicity, respectively. Intriguingly, in the last decades, the plant–human trans-kingdom migration via food intake hypothesis arose. In particular, various studies highlighted the ability of exogenous miRNAs, abundant in the mainly consumed plant-derived food, to enter the human body affecting gene expression. Notably, plant miRNAs can resist the strict conditions of the gastrointestinal tract through a methylation step that occurs during miRNA maturation, conferring high stability to these small molecules. Recent studies observed the anti-tumoral, immune modulator and anti-inflammatory abilities of trans-kingdom interaction between plant and human. Here, we depict the existing knowledge and discuss the fascinating plant–human trans-kingdom interaction, highlighting first the eventual role of plant miRNAs from foods on our somatic gene identity card and then the potential impact of using plant miRNAs as novel therapeutic avenues.

## 1. Introduction

MicroRNAs (miRNAs) are small highly conserved endogenous RNAs, with a length of about 22 nucleotides (reviewed in [1]). Starting with the discovery of lin-4 in *Caenorhabditis elegans* [2], miRNAs gained increasing interest due to their role in gene silencing at the post-transcriptional level that leads to their importance in the maintenance of a balanced physiological environment (reviewed in [3]). miRNAs are implicated in various biological functions, such as development, proliferation, differentiation, and apoptosis [4,5,6]. Currently, thousands of miRNAs have been detected in animals, plants, and microorganisms [7]. MiRNAs belonging to plants and humans share common features in term of biogenesis, function, and turnover pathways [8,9]. However, there are differences in the compartmentalization of miRNA maturation, a process occurring mainly in the nucleus in plants cells [10] and both in the nucleus and cytoplasm in human cells [11]. Moreover, in plants, a critical methylation step, lacking in humans, for miRNAs’ maturation takes place [12]. In the cytoplasm, mature miRNAs exert their function in gene silencing through mRNA target binding, which envisages full complementarity in plants [13] and a partial one in humans [14]. After target recognition, miRNAs mediate mRNA decay or translational repression [15].

Due to their role, miRNAs could be considered as part of the RNA interference (RNAi) machinery that seemed to have evolved in eukaryotes as a system of defense against viruses and transposable elements. Mobility of RNA molecules within organisms of the same species is a well-known phenomenon. In humans, an example is represented by the breast-feeding of infants, thanks to miRNA abundance in breast milk passing from mothers to the newborns [16]. Moreover, the idea that miRNAs could move between organisms of different species [17] emerged and was defined as trans-kingdom miRNA transfer [18]. This mechanism has been shown in a cross-talk between plants and microorganisms, where miRNAs act as part of the plant defense system, by migrating into the parasite cell to silence their targets, mainly linked to pests’ pathogenicity [17]. More recently, another type of trans-kingdom cross-talk has emerged: the plants-to-humans miRNAs trade via food ingestion [19]. Plant-derived foods are known to elicit various beneficial effects on human health, thanks to their composition of bioactive compounds, such as polyphenols, flavonoids, isothiocyanates, carotenoids, and others (reviewed in [20]). Since bioactive molecules exert a beneficial effect on the human body, the hypothesis that miRNAs, abundant in fruits and vegetables, might also play a pivotal role in human physiopathology through trans-kingdom mechanisms has emerged [18]. In this review, we recapitulate the current evidence on the trans-kingdom interaction between food-derived miRNAs and humans, to depict their putative role in human pathophysiology.

## 2. Human and Plant miRNAs: Analogies and Difference

**Figure 1:**
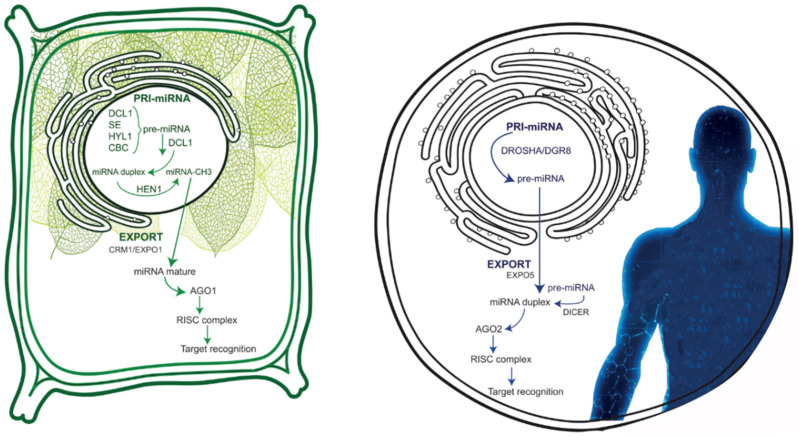
Representation of the miRNA maturation process in plant (**left**) and human (**right**). Plant miRNA maturation mainly occurs in the nucleus, where the crucial step is represented by the methylation process through the methyl transferase HEN1. Then, the plant mature miRNA is exported in the cytosol to recognize its target, after RISC complex formation. Human miRNA maturation starts in the nucleus where the pri-miRNA is cleaved to produce the pre-miRNA, which is exported in the cytosol to achieve the conclusion of miRNA maturation, then the RISC complex formation and the target recognition.

Even though it has been demonstrated that from an evolutionary perspective miRNAs are strongly conserved [21,22,23], plant and human miRNAs differ in several biogenesis and maturation steps [24,25], as well as in target recognition [13,26,27,28]. In particular, plant miRNAs bind with full complementarity to target mRNAs, resulting in cleavage and consequent degradation [13,26], while human ones are able to partially bind the 3′-UTR of the target, generally leading to the inhibition of translation [27,28]. Human miRNAs are mainly encoded by the intronic regions of both coding and non-coding genes [24] and are present in the form of a cluster [11], whereas those from plants are transcribed from intergenic regions and are rarely clustered [25,29]. Starting with the biogenesis mechanism, the first difference between human and plant miRNAs consists of the compartmentalization of the process [11]. In particular, human miRNAs are generated in the nucleus as primary miRNAs (pri-miRNAs) [11], then cleaved to produce precursor miRNAs (pre-miRNAs) [30], that are ready to be exported to cell cytoplasm in order to achieve complete maturation in the miRNA duplex. This duplex is loaded into Argonaute 2 (AGO2) protein to form the RNA-induced silencing complex (RISC) [31,32]. On the contrary, both plant miRNAs’ biogenesis and maturation take place in the nucleus, in a particular region called Dicing-bodies (D-bodies) [10]. In order to promote miRNAs’ stability and cytosolic export, plant miRNAs face a methylation step at the 2-O of the ribose on the last nucleotides at their 3′ ends [33,34]. After methylation, plant miRNA duplex goes from nucleus to the cytosol, a mechanism that has not been fully elucidated yet [35]. A recent study demonstrated that mature miRNA is loaded into Argonaute 1 (AGO1) protein to form the RISC complex and immediately exported in the cytosol via chromosomal maintenance 1/exportin1 (CRM1/EXPO1) [35]. The RISC complex, as the effector complex, is involved in target mRNAs recognition, in both humans and plants (reviewed in [36]). Between the two strands of the miRNA duplex, only the guide strand is selected in the pre-RISC complex to act in the silencing mechanism [37,38]. At this stage, miRNA is ready to target a specific mRNA by binding its 5′ end to the 3′ UTR of the target. Among the 22 nucleotides composing the sequence, only seven are in charge of target identification and form the so-called “seed region”, composed of nucleotides two to eight of the 5′ end [3]. In plants, the seed region can bind mRNA with full complementarity [13,26], while the binding in humans occurs preferentially with partial complementarity [3,27,28]. Target recognition in plants causes the cleavage and subsequently degradation of target mRNA [13,39,40], via the AGO protein of the RISC complex [41]. Human miRNAs mainly act on mRNA stability or inhibition of translation mechanism, or a combination of the two [42,43]. In order to affect target stability, miRNAs could act on polyA tail shortening or removal of the 5′ cap [44,45,46]. With regard to translational repression, miRNAs can act in the initial step [47,48] of translation as well as in the elongation step [27,49,50]. However, with translational repression, protein synthesis is inhibited, but mRNA can still be abundantly present in the cytosol [42,43,48] (Figure 1).

## 3. The Trans-Kingdom Hypothesis

Currently, the idea that miRNAs could transfer between organisms of different species has emerged and is known as “trans-kingdom”. The first evidence of this phenomenon came from the discovery of the interaction between pests and plants, enhancing pests’ pathogenicity [51] and plant resistance, respectively [52,53,54]. Interestingly, it has been demonstrated that plant miRNAs can interact with their human target via food intake, highlighting the trans-kingdom interaction between food-derived miRNAs and humans [55].

### 3.1. Trans-Kingdom in Plants and Pests

In 2013, Weiberg et al. demonstrated that *Botrytis cinerea*, a fungal pathogen causing the grey mold disease in about 200 species of plants [56], exerted its activity through miRNAs [51]. By analyzing the expression of fungal miRNAs (Bc-miRNAs) in infected plants, the authors identified three highly expressed small RNAs (Bc-siR3.1, Bc-siR3.2, and Bc-siR5) able to target plants mRNAs [51]. Using bioinformatic tools, target genes for *Botrytis cinerea* were predicted and in particular, mitogen-activated protein kinases (MAPKs) were the preferred putative targets with an implication in protection against infection in plants [51]. Specifically, Bc-small RNAs suppressed the expression of plant MAPK1 and MAPK2, which are implicated in plant immunity, peroxiredoxin (PRXIIF), which is implicated in oxidative stress, and cell wall-associated kinase (WAK) [51]. The authors demonstrated that parasite miRNAs exert a role in enhancing their pathogenicity [51]. Moreover, plants developed a mechanism to improve resistance to pests infection through RNAi machinery [52,53,54]. Mao and colleagues elucidated the process used by the herbivore pest *Helicoverpa armigera* to cause yield loss mainly in cotton and other crops [57]. Cotton plants store gossypol, a toxic metabolite for the majority of organisms, in their pigmented glands [58,59]. *Helicoverpa armigera* is able to resist this toxic molecule through the cytochrome P450 gene CYP6AE14, which is present at high levels in the mid gut and implicated in gossypol catabolism, improving larval growth and infection spreading [57]. Plant expression of a double stranded RNA (dsRNA) targeting CYP6AE14 led to cytochrome downregulation and larval growth inhibition, proposing an important role for plant RNAi pathways in the defense against parasites and offering a useful alternative to common insecticides and pesticides [57,60]. A similar trans-kingdom cross-talk has been highlighted between humans and the pathogenic factor of malaria, *Plasmodium falciparum* [61]. LaMonte et al. analyzed the miRNA profile of erythrocytes with mutant allele (HbS) that confers major resistance to *Plasmodium falciparum* [62], showing an up-regulation of human miR-451 (hsa-miR451) during malaria infection, only in mutant erythrocytes [63]. This miRNA translocates into the parasite, generating a fusion with cAMP-dependent protein kinase (PKA-R) of *Plasmodium falciparum* mRNA, that leads to erythrocyte resistance to malaria infection [63]. In fact, transfection of miR-451 in erythrocytes conferred resistance to *Plasmodium Falciparum*, indicating the crucial role of miR-451 in parasite growth control [63].

### 3.2. Trans-Kingdom Between Food-Derived miRNAs and Humans in Health and Diseases

Since plant-derived food and human cells are rich in miRNAs [64], the hypothesis about the ability of food-derived miRNAs to transfer within human body has emerged [55]. An intriguing piece of this fascinating puzzle is the passage of plant miRNAs from breastfeeding mothers to their infants. Human milk contains both endogenous miRNAs, able to pass to the offspring [16,65], and exogenous plant miRNAs. Although endogenous miRNAs have been detected at higher levels than exogenous ones, plant miR168a, miR156a, and miR166a were the most abundant in milk samples collected from breast feeding women [66]. Further investigations are needed to validate the presence of these exogenous miRNAs in the breastfed infants [66].

In addition, plant miRNAs are also present in juice derived from fruit [67]. To assess the presence of exogenous miRNAs in human serum, nine healthy volunteers consumed a fruit mix or drank watermelon juice [67]. In the sera of these subjects, 10 plant miRNAs were identified, with a peak of expression three hours after administration of either fruit mix or watermelon juice [67].

**Figure 2:**
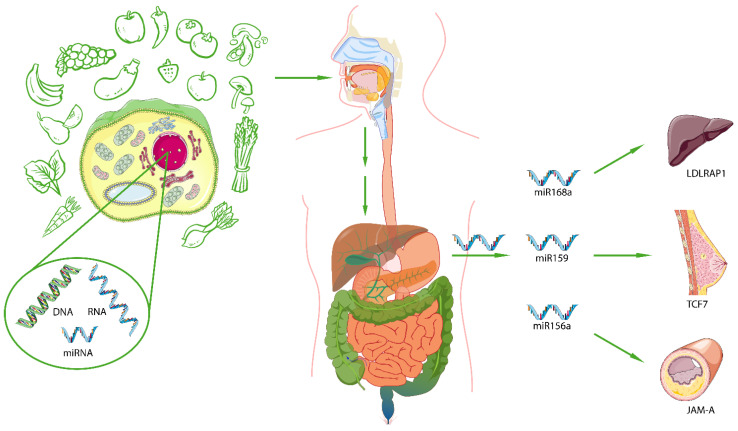
Role of plant miRNAs on human pathophysiology. Plant-derived foods are rich in miRNAs, which are able to resist the strict environment of the human gastrointestinal tract. After being absorbed in the intestine, exogenous miRNAs move to the blood circulation and reach their target in human cells. In particular, miR168a, highly expressed in rice, is able to inhibit the low-density lipoprotein receptor adapter protein 1 (LDLRAP1) in the liver. MiR159, isolated from broccoli, seems to be inversely related to breast cancer incidence and progression, by binding to the transcription factor 7 (TCF7). Plant (green vegetables) miR156a has an atheroprotective role through the inhibition of the junctional adhesion molecule-A (JAM-A) involved in the first step of the formation of the atherosclerotic plaques.

Interestingly, the peculiar plant miRNAs methylation at the 3′ terminus led Zhang et al. to identify about 30 exogenous miRNAs in the serum of healthy Chinese subjects [19]. miR168a is particularly present in rice, which is the predominant food in the Chinese diet, and represents one of the most highly enriched plant miRNAs in the sera of Chinese subjects [19]. In vitro and in vivo experiments showed that miR168a is able to bind the low-density lipoprotein receptor adapter protein 1 (LDLRAP1) mRNA, inhibiting its expression in the liver. This study indicated that exogenous plant miRNAs, taken with foods, could modulate the expression of target genes in humans [19] (Figure 2).

Furthermore, many studies have been conducted to further demonstrate the miRNAs trans-kingdom across plants and humans [66,67,68,69]. Several studies have illustrated further insights on the effects of plant-derived miRNAs on mammalian cells, highlighting the involvement of exogenous miRNAs in the pathogenesis of human diseases as anti-viral [70,71], anti-tumoral [68,72,73,74,75,76], immune modulating [75], and anti-inflammatory [69,77,78] molecules.

miR159, a miRNA with a conserved sequence between different plant species, such as *Arabidopsis thaliana*, *Glycine max* (soybean), and *Brassica oleracea* (broccoli), has been detected in sera of healthy women and patients with breast cancer (BC) at different stages [68]. Interestingly, a reduced expression of miR159 in BC patients compared to controls has been observed, showing an inverse correlation between the level of exogenous miRNA and BC onset and progression, and it has been associated to a negative status of the estrogen and progesterone receptors (ER and PR, respectively) [68]. In silico analysis uncovered three putative miRNA targets in humans [68], namely the transcription factor 7 (TCF7) [79], nuclear receptor coactivator 6 (NCoA6) [80], and engrailed-2 (EN2) [81], all upregulated in BC and exploiting their role in tumor initiation and progression [79,80,81]. In particular, TCF7, a transcription factor involved in the Wnt/β-catenin pathway [79], showed the major complementarity to exogenous miR159 [68]. In vitro and in vivo analyses revealed that synthetic miR159, when used to transfect BC cell lines or administered to xenograft mouse models, was able to reduce TCF7 levels and consequently, its downstream target, c-MYC [82] (Figure 2). In addition, human colorectal carcinoma cell lines Caco-2 transfected with miR159a extracted from soybean seeds displayed decreased TCF7 expression, thus leading to a reduction in cellular proliferation [73].

*Lonicera japonica*, better known as honeysuckle, has been used in infusion since ancient times in Asia to counteract influenza symptoms [83,84]. Interestingly, honeysuckle miRNA, miR2911, is able to resist high temperatures used during the boiling necessary for infusion preparation, thanks to the high content of CG basis in its sequence [70,71]. The role of miR2911 has been evaluated in fighting human viruses, with particular attention to the Influenza A virus and SARS-CoV-2 [70,71]. In mouse models, the administration of honeysuckle decoction increased the expression of exogenous miR2911 in the blood. Moreover, administration of mir2911 or honeysuckle decoction decreased mouse mortality caused by Influenza A infection thanks to the ability of miR2911 to target viral genes involved in virus replication [70]. In addition, bioinformatic analysis revealed that miR2911 is able to bind genes involved in SARS-CoV-2 replication [70]. In particular, honeysuckle decoction administered to patients with moderate SARS-CoV-2 infection led to inhibition of virus replication, thus facilitating patients’ virus negativization.

Further examples of trans-kingdom cross-talk have been investigated between the plants, *Moringa oleifera* and *Olea europaea,* and human cell lines. In particular, miRNAs derived from *Moringa oleifera* (mol-miRNAs) are able to target human interleukin 2 receptor subunit alpha (IL2RA), tumor necrosis factor (TNF) and Vav Guanine Nucleotide Exchange Factor 1 (VAV1), which are mainly involved in cell cycle regulation, apoptosis, immune response and HIV pathogenesis [85,86,87,88]. In vitro analysis on HIV-infected Peripheral Blood Mononuclear Cells (PBMCs) transfected with mol-miRNAs exhibited a decrease in viability related to increased apoptosis, an increase in T helper cells and a downregulation of VAV1 [75]. On the contrary, PBMCs from healthy donors were not affected by mol-miRNAs transfection, suggesting a putative role for *Moringa oleifera* and its miRNAs as novel therapeutic molecules in the treatment of viral infection [75]. Additionally, *Moringa oleifera* has been investigated for its anti-tumoral properties. Potestà et al. performed bioinformatic analysis to identify human putative targets for miRNAs in *Moringa oleifera* microvescicles and identified their pro-apoptotic role in targeting the mRNA of the anti-apoptotic B-cell lymphoma 2 (BCL-2) [76], which is dysregulated in the majority of tumors with epithelial origin and is also involved in maintaining mitochondrial membrane potential [89,90]. To prove the pro-apoptotic potential of mol-miRNAs, miRNAs-enriched microvescicles were added to cell cultures of tumor cell lines demonstrating an enhanced apoptosis, a decrease in BCL-2 and a change in mitochondrial membrane potential [76]. Additionally, transfection of porcine jejunum epithelial cell lines (IPEC-J2) and Caco-2 cells with mol-miRNAs showed a reduction in cell proliferation and a consistent reduction in β-catenin, which is implicated in colorectal cancer [72]. Down-regulation of β-catenin consequently led to downregulation of downstream target genes, such as the oncogene c-MYC and Proliferating Cell Nuclear Antigen (PCNA) [72]. The anti-tumorigenic effect of plant miRNAs has also been highlighted in *Olea europaea* miRNAs (oeu-miRNAs). First, in silico analysis revealed that these miRNAs shared high similarity with a human miRNA involved in tumor inhibition, hsa-miR-34a, suggesting that they have the same target transcript [74,91]. Transfection of oeu-miRNAs in hsa-miR34a-deficient cell lines and PBMCs from healthy donors showed that oeu-miRNAs are able to restore cells functions mediated by hsa-miR34a, even in cells lacking this miRNA, while having no effect in PBMCs where miRNA expression is normal [74]. These data highlight the possibility that plant-derived miRNAs with functional homology to human miRNAs could support anti-tumoral strategies.

Furthermore, it has been hypothesized that miRNAs derived from fruit and vegetables could be involved in the protection against cardiovascular diseases (CVD) [20]. In this respect, miR156a, abundant in green vegetables, particularly cabbage, spinach, and lettuce, was detected at higher levels in the serum of control subjects in comparison to patients with CVD [69], suggesting its putative role in the protection against atherosclerosis. Bioinformatic analysis of miR156a target genes revealed junctional adhesion molecule-A (JAM-A) as a putative target [69] (Figure 2). JAM-A is a gap junction protein involved in the inflammatory recruitment of monocytes to the endothelial of vessels and its accumulation is seen in the early stage of the formation of the atherosclerotic plaques [92]. To investigate the link between plant miR156a and human JAM-A, human aortic endothelial cells (HAECs) were transfected with the miR156a, which resulted in a significant reduction in JAM-A levels [69], demonstrating an innovative vasoprotective molecular mechanism of green vegetables through plant microRNAs (Table 1).

Finally, preclinical studies using animal miRNAs with a role in cancer onset and progression, have been performed in order to identify the optimal administration strategy with minimal side effects [93,94,95,96,97,98,99,100]. miR-34a mimic encapsulated in lipid nanoparticles and have been investigated in phase I clinical trial on patients with advanced solid tumors (NCT01829971) [101], due to the important results reached in preclinical studies in mouse models of lung cancer [96,100]. Indeed, mouse models of non-small cell lung cancer, characterized by a tissue-specific heterozygosis mutation of the *KRAS* gene, displayed a reduction in the area of the pre-formed tumors after miR-34a administration via tail-vein injection or via a tracheal catheter [96,100]. Additionally, in mice, plant-derived nanoparticles containing miRNAs [102] have been shown to act mainly on intestinal stem cells of the crypt, promoting intestinal tissue renewal and providing protection against DSS-induced colitis [103,104].

## 4. Future Perspectives

In the last decades, the idea that miRNAs could move across organisms of different species emerged under the name of “trans-kingdom”. Although the first evidence came from a mechanism of defense against pests and parasites in both plants and animals, the trans-kingdom hypothesis has been extended to plants and humans. In particular, plant miRNAs could act on the human body via food intake, having a beneficial effect on human health. The relevance of miRNAs in human pathophysiology makes them significant candidates for the development of therapeutic or nutraceutic approaches in the treatment of various human diseases. Emerging evidence strongly points to miRNA exploitation for a wide range of clinical applications due to the fact that they can be observed in bodily fluids, could be used as biomarkers, and regulate inflammatory, metabolic, and proliferative pathways in health and diseases. As the field of trans-kingdom miRNA research advances, the therapeutic and nutraceutic value of miRNAs continues to increase. In particular, the current knowledge about the abundant presence of miRNAs in plants and in plant-derived foods and their resistance even to the high temperature in the case of cooked foods, make them of great interest in the field of food and agriculture. Specific attention could be given to the peculiar miRNAs of the most consumed plants and their implication not only in human health but also in plant growing and development. This could be the starting point to develop new strategies in agriculture, to implement miRNAs amount in fruit and vegetables, to have benefit in the plant production and healthy food consumption. Moreover, plant-based miRNAs are now under investigation for a wide array of human pathologies, ranging from inflammation to metabolism and cancer, offering the advantages of high specificity and fewer adverse effects and toxicity. 

## Figures and Tables

**Table 1:** In vivo and in vitro studies demonstrating the plant–human trans-kingdom.

**miRNA source**	**miRNA**	**Results**	**Ref.**
**In vivo**			
Plant: rice	miR-168a	Exogenous miR-168a presence in sera from healthy Chinese women and men. Exogenous miR168a detection in sera and liver from mice fed a diet enriched in rice after 12 h. LDLRAP1 expression reduction in vitro and in vivo via exogenous miR-168a.	Zhang et al., 2012 [19]
Plant: common dietary fruit and vegetables	miR-168a miR-156a miR-166a miR-172a miR-167a	Presence of exogenous miRNAs in breast milk of volunteers with different dietary patterns. miR-156a and miR-168a abundance in the exosomes fraction from breast milk.	Lukasik et al., 2017 [66]
Plant: fruit mix and watermelon juice	miR-156a miR-177a miR-162a miR-390a miR-168a	MiRNAs identification in sera of healthy volunteers with a peak of abundance 3 h after fruit mix or watermelon juice ingestion.	Liang et al., 2015 [67]
Plant: *Arabidopsis thaliana*, soybean, broccoli	miR-159	miR-159 different abundance in sera of healthy women compared with BC patients at different stages. Decrease in tumor growth in vitro and in vivo in xenograft mouse models via miR-159 administration. Transcription factor TCF7, mainly upregulated in BC, reduction after miR-159 transfection.	Chin et al., 2015 [68]
Plant: honeysuckle	miR-2911	miR-2911 abundance in honeysuckle decoction. Influenza A and SARS-CoV-2 genes involved in viral replication predicted target for miR-2911. Reduction in mortality rate of mice with Influenza A virus infection after miR-2911. Negative conversion promotion in COVID-19 patients after miR-2911 additional treatment	Zhou et al., 2015 [70] Zhou et al., 2020 [71]
Plant: green vegetables (cabbage, spinach, and lettuce)	miR-156a	miR-156a differently presence in sera of healthy donor and CVD patients. Gap junction JAM-A identification as a target for miR-156a. Reduction in atherosclerotic plaque formation in vitro through miR-156a transfection.	Hou et al., 2018 [69]
**miRNA source**	**miRNA**	**Results**	**Ref.**
**In vitro**			
Plant: *Moringa oleifera*	miR-858b	Viability of HIV-infected-PBMCs decrease via VAV1 downregulation. Reduction in CD4 T cell activation and differentiation after miR-858b transfection.	Minutolo et al., 2020 [75]
Plant: *Moringa oleifera*	miRNAs from microvesicles	Pro-apoptotic role in tumor cell lines of mol-miRNAs. B-cell lymphoma 2 (BCL-2) as exogenous miRNAs predicted target gene. BCL-2 downregulation, change in the mitochondrial membrane, and increase in apoptosis after mol-miRNA transfection.	Potesta et al., 2020 [76]
Plant: *Moringa oleifera*, soybean, wheat, maize	miR-167e-5p	Intestinal cell lines proliferation reduction via miR-167e-5p. Downregulation of β-catenin expression by miR-167e-5p binding.	Li et al., 2019 [72]
Plant: *Olea europaea*	miR-27 miR-34	In silico identification of *O.europaea* miRNAs and similarity with hsa-miR-34a-5p. oeu-miRNAs and has-miR-34a-5p target annotation. Implication in tumorigenesis of human cell lines for oeu-miRNAs.	Minutolo et al., 2018 [74]
